# Molecular Characterization of *Listeria monocytogenes* Isolated from Retail Yak Meat in Nyingchi, Xizang, China

**DOI:** 10.3390/ani16172729

**Published:** 2026-09-02

**Authors:** Jiaojiao Xin, Yongzhi Lou, Sizhu Suolang, Yuelan Yin, Xinan Jiao, Bujie Duo, Zhuoga Baima, Ga Gong

**Affiliations:** 1 College of Animal Science, Xizang Agricultural and Animal Husbandry University, Nyingchi 860000, China; sxyaxj@163.com (J.X.); hnrzlyz@163.com (Y.L.); xzslsz@163.com (S.S.); 2 Jiangsu Key Laboratory of Zoonosis, Yangzhou University, Yangzhou 225009, China; yylan@yzu.edu.cn (Y.Y.);; 3 Damxung County Veterinary Station, Lhasa 851500, China

**Keywords:** *Listeria monocytogenes*, yak meat, Xizang, whole-genome sequencing (WGS), virulence genes

## Abstract

*Listeria monocytogenes* is a Gram-positive zoonotic pathogen responsible for listeriosis, a severe foodborne disease with high mortality in humans and animals. This study aimed to investigate the molecular epidemiology and genomic characteristics of *L. monocytogenes* isolated from raw yak meat in Nyingchi, Xizang, China. A total of 231 yak-related samples were collected in Nyingchi, consisting of 214 retail raw yak meat samples, 14 farm environmental samples, and 3 nearby water source samples. *L. monocytogenes* isolates were identified and characterized using culture-based methods, PCR serotyping, and whole-genome sequencing (WGS). Bioinformatic analyses were performed for virulence, antimicrobial resistance, and functional gene annotation using KEGG and COG databases. The overall contamination rate of Lm was 13.08% (28/214) for retail raw yak meat samples, whereas no isolates were recovered from 14 farm environmental samples (0.00%, 0/14) and 3 nearby water source samples (0.00%, 0/3). The serotypes of isolates were 1/2a (9/28, 32.14%), 1/2b (7/28, 25.00%), and 1/2c (12/28, 42.86%). These 28 isolates exhibited varied antimicrobial resistance profiles, with universal resistance to trimethoprim-sulfamethoxazole, high resistance to erythromycin and clindamycin, and low resistance to vancomycin. MLST analysis revealed seven sequence types (STs): ST9 (12/28, 42.86%), ST619 (6/28, 21.43%), ST8 (6/28, 21.43%), ST7 (1/28, 3.57%), ST87 (1/28, 3.57%), ST121 (1/28, 3.57%), ST297 (1/28, 3.57%). ST619 isolates harbored multiple virulence genes, including those located on Listeria pathogenicity islands LIPI-1, LIPI-3, and LIPI-4, indicating high genomic potential for virulence. Representative isolate Y2 (ST619) possessed a 3,009,858 bp genome with 3036 coding genes, four genomic islands, and two prophages. Functional annotation revealed enrichment of genes involved in carbohydrate transport and metabolism and amino acid biosynthesis pathways. Our findings provide the first genomic insight into *L. monocytogenes* contamination in yak meat from Nyingchi, Xizang, China, highlighting the urgent need to strengthen food safety monitoring and hygiene management in this region.

## Simple Summary

Yaks represent a vital component of the ecological system on the Qinghai–Tibet Plateau of China. Their meat products hold significant value for local herdsmen. To elucidate the epidemiological characteristics of *Listeria monocytogenes* isolated from yaks in Nyingchi, Xizang, China, we investigated this pathogen in fresh yak meat retail outlets and surrounding environments of yak farms, and performed whole-genome sequencing on the representative isolate Y2 (ST619). Our results revealed an isolation rate of 13.08% (28/214) for *Listeria monocytogenes* from yak meat, with high diversity in virulence, antimicrobial resistance and molecular genetics. This study highlights the potential risks of environmental persistence and cross-contamination of *Listeria monocytogenes* during yak meat processing. Targeted monitoring of its contamination sources is essential to reduce the risk of human infection.

## 1. Introduction

*Listeria monocytogenes* is a ubiquitous Gram-positive, facultatively intracellular pathogen capable of infecting both humans and a diverse range of animals [1]. It is the causative agent of invasive listeriosis, a severe foodborne disease with a high mortality rate (20%~30%) [2]. Ruminants are particularly susceptible to *L. monocytogenes* infection, often serving as significant reservoirs [3,4,5,6,7,8,9]. Infected animals can shed the pathogen into the environment via feces, facilitating its transmission throughout the food production chain [10]. Consequently, *L. monocytogenes* contamination in animal-derived products represents a major challenge to global food safety and public health. Although raw yak meat is generally fully cooked before consumption, *L. monocytogenes* originating from meat can cause cross-contamination during retail circulation and household food handling. The pathogen can spread to ready-to-eat foods and threaten immunocompromised people, which makes meat-borne listeriosis surveillance indispensable [11].

China is the primary center of origin and genetic diversity for the yak (*Bos grunniens*), the dominant livestock species on the Tibetan Plateau [12]. Yak meat is a staple protein source for local populations and is increasingly being exported to other regions. In recent years, yak husbandry has undergone a paradigm shift from traditional extensive grazing to large-scale intensive farming to meet growing market demands [13]. This intensification has been accompanied by an increase in zoonotic bacterial infections among yaks [14]. While numerous studies have documented *L. monocytogenes* prevalence in cattle and sheep globally, there is a significant knowledge gap regarding *L. monocytogenes* in yak meat within high-altitude environments [15,16,17]. Specifically, the molecular characteristics, virulence potential, and evolutionary dynamics of *L. monocytogenes* strains circulating in the Tibetan yak meat chain remain poorly understood.

Molecular typing based on whole-genome sequencing (WGS) has revolutionized the epidemiological surveillance of foodborne pathogens [18]. Unlike traditional methods such as pulsed-field gel electrophoresis (PFGE), WGS offers unparalleled resolution for differentiating strains [19]. Currently, multilocus sequence typing (MLST) is a widely adopted typing scheme to assign sequence types (STs) to *L. monocytogenes* isolates and support ST-based comparison between different strains [20]. To achieve finer discrimination, core genome MLST (cgMLST), which analyzes 1748 loci, is increasingly employed to decipher micro-evolutionary relationships and explore possible genetic relatedness and contamination patterns among isolates [21]. Furthermore, comparative genomic analyses, including synteny and phylogenomic clustering, are critical for identifying virulence determinants and tracking the source of contamination in the food chain [22].

Given the unique ecological conditions of the Tibetan Plateau and the specificities of yak husbandry, investigating *L. monocytogenes* contamination is essential for regional food safety. In this study, we systematically evaluated *L. monocytogenes* prevalence in the Nyingchi region of Xizang. Specifically, this study aimed to: (i) assess the contamination rate and molecular diversity of *L. monocytogenes* in the yak meat chain; (ii) determine the pathogenic and resistance potential of these isolates; and (iii) elucidate the phylogenetic relationships between Tibetan strains and global isolates. Our findings provide a scientific framework for listeriosis prevention and support the development of enhanced food safety management strategies in high-altitude regions.

## 2. Materials and Methods

### 2.1. Study Design and Sample Collection

**Figure 1:**
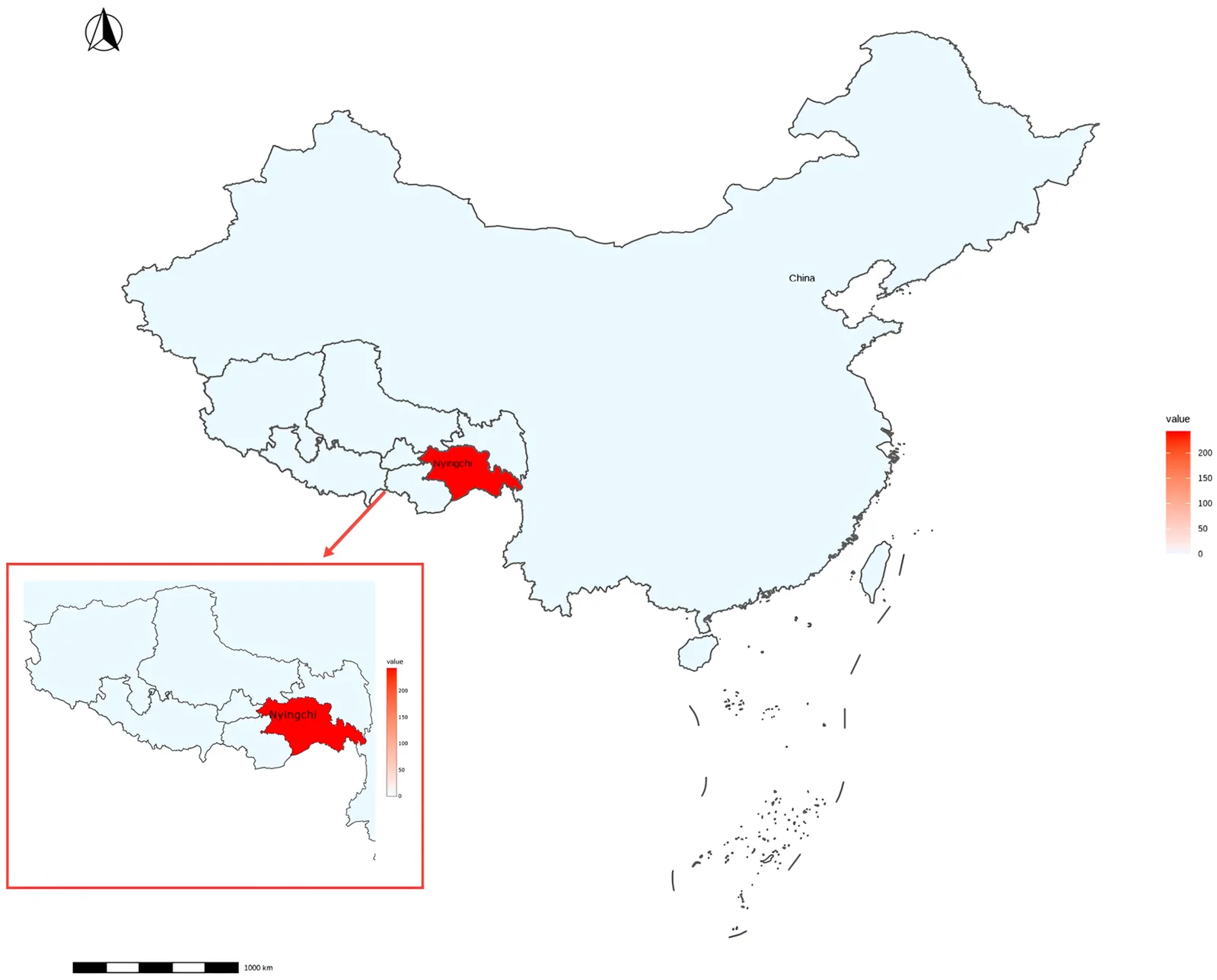
Geographical distribution of sample collection sites (samples were collected in Nyingchi, Xizang, China).

All samples were collected in the autumn of 2023–2024, specifically October 2023, August and September 2024. In total, 231 yak-associated meat and environmental samples were obtained from four markets, 79 retail stalls, two farms, and two water sources in Nyingchi, Xizang, China. The number of samples collected at each sampling site varied, and 19 out of the 87 sampling sites tested positive for the target pathogen (Figure 1). Sampling was conducted randomly, with each meat sample consisted of approximately 25 g of fresh yak skeletal muscle. Meanwhile, environmental samples were collected from farm floors, feeding utensils, and surface water using sterile cotton swabs pre-moistened with transport medium. All specimens were collected under strict aseptic conditions, with convenience sampling adopted throughout the investigation. After collection, all specimens were immediately stored at 4 °C in a vehicle-mounted refrigerator and transported to the Preventive Veterinary Medicine Laboratory of Xizang Agricultural and Animal Husbandry University. Isolation and molecular identification of *L. monocytogenes* were initiated within 12 h of sampling. Every specimen was analyzed individually, and no pooled samples were used in this study.

### 2.2. L. monocytogenes Isolation and Identification

Isolation was performed according to the US Department of Agriculture-Food and Safety (USDA-FSIS) method for *L. monocytogenes* [23]. Briefly, all samples were mixed with University of Vermont Medium (UVM; Becton, Dickinson and Company, Franklin Lakes, NJ, USA) at a 1:9 sample-to-broth dilution ratio. The mixture was incubated statically at 30 °C for 24 h for primary enrichment. Following primary enrichment, 0.1 mL of the UVM culture was transferred to 10 mL of Fraser broth (Becton, Dickinson and Company, Franklin Lakes, NJ, USA) for secondary enrichment, followed by static incubation at 37 °C for 24 h. The enriched samples were then streaked onto CHROMagar™ Listeria (CHROMagar Company, Paris, France) and incubated at 37 °C for 18 h to facilitate colony isolation.

Three presumptive colonies from each sample were purified on brain–heart infusion (BHI; Solarbio, Beijing, China) agar plates at 37 °C for 18 h. All purified isolates were first subjected to species-level identification by species-specific PCR with published primer sequences [24]. All isolates testing positive were further serotyped following the method described by Doumith et al. [25]. Isolates with identical serotypes from the same sample were reduced to one representative strain.

### 2.3. Genomic DNA Extraction and Genome Sequencing

Genomic DNA was extracted from 28 *L. monocytogenes* isolates using a bacterial genomic DNA extraction kit (Tiangen Biotech, Beijing, China). DNA quality was determined with a NanoDrop™ 2000 spectrophotometer (Thermo Scientific, Waltham, MA, USA). Only samples with a DNA concentration ≥ 50 ng/μL, an A260/A280 ratio of 1.8–2.0, and an A260/A230 ratio > 1.5 were subjected to subsequent sequencing. Qualified DNA samples were sent to Sangon Biotech (Shanghai, China) for library preparation and whole-genome sequencing. Libraries were performed using the Illumina DNA Prep Kit for Illumina (Illumina, San Diego, CA, USA). Whole-genome sequencing of all isolates was performed on the Illumina HiSeq platform with a 2 × 150 bp paired-end strategy, yielding an average sequencing depth of >100×. All sequencing datasets met the following quality thresholds: ≥95% genome coverage, ≥98% genic region coverage, and a Q30 base quality proportion of ≥85%. Raw reads were quality-filtered using Fastp v0.20.0 (Q < 20), and subsequently de novo assembled using SPAdes v3.15.0. Assembly quality was evaluated with QUAST v5.0.2. Additionally, genome completeness and contamination were evaluated using Kraken 2 v2.1.2, BUSCO v5.4.3, CheckM v1.2.0, and ContEst 16S.

Furthermore, one representative yak-derived *L. monocytogenes* isolate (Y2, ST619) was further subjected to third-generation long-read sequencing on the PacBio Sequel platform (Pacific Biosciences, Menlo Park, CA, USA) for complete genome assembly. This isolate was prioritized due to its extensive repertoire of antimicrobial resistance genes and virulence determinants. The genome sequence of Y2 has been deposited in GenBank under accession number CP184647. The remaining 27 draft genomes were generated exclusively for in-house comparative genomic analysis in this study and have not been deposited in a public database.

### 2.4. Genomic and Bioinformatic Analysis

Multilocus sequence typing (MLST) was performed using the *L. monocytogenes* BIGSdb 1.51.0 (https://bigsdb.pasteur.fr/listeria/) (accessed on 3 November 2025) database based on seven housekeeping genes. Virulence genes were identified using the Virulence Factor Database (VFDB 2025, https://www.mgc.ac.cn/VFs/search_VFs.htm) (accessed on 10 November 2025) with alignment thresholds of >90% identity and >85% coverage. Antimicrobial resistance (AMR) genes were annotated using the Comprehensive Antibiotic Resistance Database (CARD 4.0.1, https://card.mcmaster.ca/home) (accessed on 10 November 2025) with BLAST-based searches (identity and coverage thresholds set at 80%) (https://blast.ncbi.nlm.nih.gov/, accessed on 10 November 2025).

Predictions of coding sequences (CDS), tRNA, and rRNA genes were conducted using NCBI-PGAP 1.2.1 and Prokka 1.10 pipelines. Functional annotation was carried out by assigning predicted genes to the Gene Ontology (GO, http://www.geneontology.org/), Kyoto Encyclopedia of Genes and Genomes (KEGG, http://www.kegg.jp), and Clusters of Orthologous Groups (COG, https://www.ncbi.nlm.nih.gov/COG/, accessed on 21 November 2025) databases. Additionally, genes were annotated for carbohydrate-active enzymes using the CAZy database.

Structural features of proteins were analyzed using dedicated bioinformatics tools: Membrane and signal peptide structures were predicted using TMHMM 2.0 and SignalP 4.1, respectively, while lipoproteins were detected using LipoP 1.0. Subcellular localization of proteins was determined by ProtComp 9.0. Genomic islands were identified using IslandPath-DIOMB 0.2. Prophages were detected using PhiSpy 2.3, and predictions were further validated via BLAST alignment against known phage sequences from PHAST 1.0.

### 2.5. Comparative Genomic Analysis

cgMLST was performed utilizing the Pasteur Institute scheme (1748 loci). Based on the resulting profiles, minimum spanning trees (MSTs) were derived using GrapeTree 1.3.5, with subsequent visualization performed on the Interactive Tree of Life (iTOL) platform.

To explore the evolutionary background of isolate Y2, homologous genome sequences were retrieved from the NCBI nucleotide (NT) database. Thirty representative *L. monocytogenes* genomes with complete annotations were selected for comparative analysis. The 16S rRNA sequences of all strains were aligned using ClustalW 2.1, and a phylogenetic tree was constructed.

### 2.6. Antimicrobial Susceptibility Testing

Antimicrobial susceptibility testing of all *L. monocytogenes* isolates was performed using the Kirby–Bauer (K–B) disk diffusion method, in compliance with the European Committee on Antimicrobial Susceptibility Testing (EUCAST) Disk Diffusion Method for Antimicrobial Susceptibility Testing v. 13.0 (2025). Inhibition zone diameters were measured and interpreted according to two sets of standard criteria: the EUCAST Breakpoint Tables for Interpretation of Zone Diameters v. 16.0 (2026), and the Clinical and Laboratory Standards Institute (CLSI) M100 Ed33 (2023) performance standard for antimicrobial susceptibility testing [26,27,28].

Briefly, the turbidity of the bacterial suspension was adjusted to the 0.5 McFarland standard. Subsequently, the prepared suspension was evenly spread onto Mueller–Hinton agar plates (Condalab, Madrid, Spain) with 5% defibrinated horse blood and 20 mg/L β-NAD (MH-F). Within 15 min of inoculation, antimicrobial disks were applied to the agar surface and spaced sufficiently to avoid overlapping inhibition zones. All plates were incubated at 35 ± 1 °C for 18 ± 2 h in an atmosphere of 5% CO_2_. Inhibition zone diameters were measured in millimeters. All antimicrobial disks were purchased from Hangzhou Microbial Reagent Co., Ltd., Hangzhou, China. The antimicrobial disk potencies are provided in Table 1.

No species-specific interpretive breakpoints for *L. monocytogenes* are specified in current CLSI standards for nitrofurantoin, clindamycin, ciprofloxacin, vancomycin, tetracycline, gentamicin, and chloramphenicol. Accordingly, susceptibility results for these agents were interpreted using *Staphylococcus* spp. breakpoints from CLSI M100 Ed33 (2023), consistent with standard practice in global *L. monocytogenes* resistance surveillance and peer-reviewed studies [29,30]. Of note, these results are intended exclusively for epidemiological comparative analysis and do not guide clinical antimicrobial therapy. The isolates were categorized as resistant (R), intermediate (I), or susceptible (S) (Table 1). Three reference strains (*Staphylococcus aureus* ATCC 29213, *Streptococcus pneumoniae* ATCC 49619, *Escherichia coli* ATCC 25922) were run as quality controls for each test batch. All measured zone diameters complied with the official quality control ranges defined in CLSI M100 Ed33 (2023), validating the consistency and reliability of the entire assay.

### 2.7. Biofilm Formation Assay

Biofilm-forming ability was quantified via crystal violet (CV) staining. The experiment was performed following the method reported by Stepanović, S [31]. Briefly, bacterial cultures were grown to an OD_600_ of approximately 0.2, diluted 1:100 in tryptic soy broth (TSB; Becton, Dickinson and Company, Franklin Lakes, NJ, USA), and added (200 µL/well) into sterile 96-well polystyrene microplates. After incubation at 37 °C for 24 h, the wells were washed three times with PBS, fixed with methanol for 15 min, and stained with 2% CV for 5 min. Excess stain was removed by washing with sterile distilled water, and the bound dye was solubilized with 33% glacial acetic acid. The optical density (OD_570_) was measured to quantify biofilm biomass. Each strain was tested in triplicate with negative and blank controls included on each plate. Each strain was assayed in triplicate. These three parallel measurements represented technical replicates. Negative and blank controls were loaded on each plate.

In short, the cut-off optical density (ODc) was defined as the mean OD value of the NC plus three standard deviations (ODc = mean OD of NC + 3 × SD). Biofilm-forming capacity was classified based on the following criteria: non-adherent (OD ≤ ODC); weakly adherent (ODC < OD ≤ 2 × ODC); moderately adherent (2 × ODC < OD ≤ 4 × ODC); strongly adherent (OD > 4 × ODC).

### 2.8. Statistical Analysis

For biofilm formation assays, intergroup differences across distinct sequence types and sampling years were analyzed separately. Differences among experimental groups were evaluated using one-way analysis of variance (ANOVA), followed by Tukey’s post hoc test for multiple comparisons test. For all tests, *p* < 0.05 indicates a statistically significant difference. Statistical analyses and graphing were conducted with GraphPad Prism version 9.0.0.

## 3. Results

### 3.1. Prevalence and Molecular Typing of L. monocytogenes from Yak Meat

Among the 231 samples collected from the Nyingchi region, 28 (12.12%) tested positive for *Listeria monocytogenes*. The contamination rate was 13.08% (28/214) in raw yak meat samples. Significantly, only one environmental sample (from 17 farm swabs and water samples) was positive, and this isolate was a non-pathogenic Listeria sp. These findings indicate that raw yak meat constitutes the main positive sample category in this survey of *L. monocytogenes* in the surveyed region.

**Figure 2:**
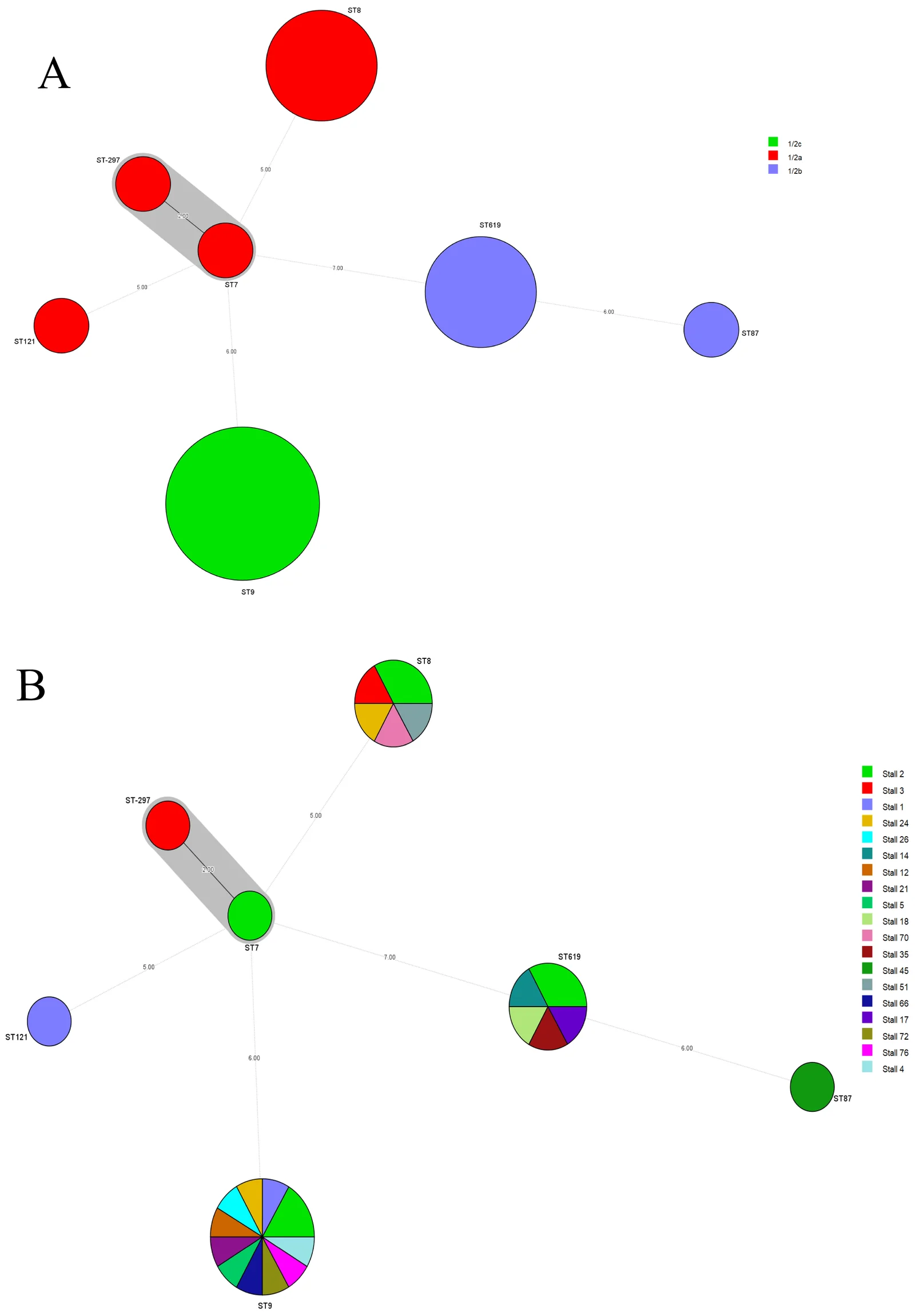
Minimum spanning tree (MST) based on MLST profiles of Lm isolates. Circle size indicates the number of isolates, and colors represent the serotypes (**A**) and retail stall origins (**B**) of the isolates (Detailed information including exact ST, serotype, corresponding stall origin, and isolate number for each subgroup is fully provided in Supplementary File S1.

The 28 *L. monocytogenes* isolates were grouped into three major serotypes: 1/2a (9/28, 32.14%), 1/2b (7/28, 25.00%) and 1/2c (12/28, 42.86%). MLST analysis showed seven sequence types (STs): ST9 (12/28, 42.86%), ST619 (6/28, 21.43%), ST8 (6/28, 21.43%), ST7 (1/28, 3.57%), ST87 (1/28, 3.57%), ST121 (1/28, 3.57%), ST297 (1/28, 3.57%). Of note, ST619 isolates were exclusively associated with the 1/2b serotype and originated from retail yak meat samples (Figure 2A).

*L. monocytogenes* contamination was detected in 19 retail stalls, with stall 2 yielding the highest number of *L. monocytogenes* isolates (Figure 2B). Multiple identical genotypes were identified among isolates from different stalls, suggesting that these yak meat products may originate from a common source.

### 3.2. L. monocytogenes Isolates from Yak Meat Exhibited the Presence of Virulence-Associated Genomic Determinants

**Figure 3:**
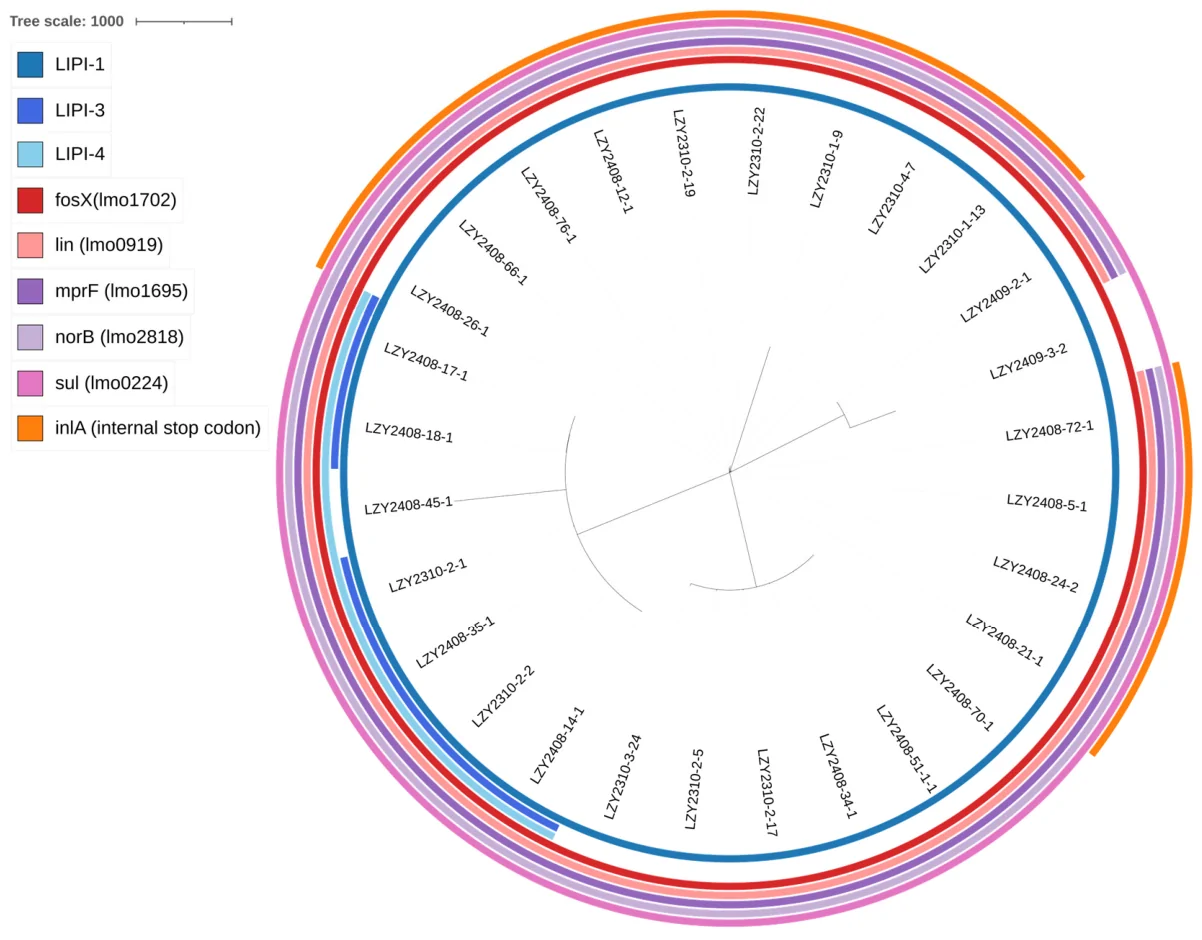
Analysis of genomic characteristics of *L. monocytogenes* strains from yak meat. Branches based on cgMLST were shown in the center of the circular plot. Each strain was labeled for: (1) virulence genes within pathogenicity islands LIPI-1 (*prfA*, *plcA*, *hly*, *mpl*, *actA*, *plcB*), LIPI-3 (*llsA*, *llsG*, *llsH*, *llsX*, *llsB*, *llsY*, *llsD*, *llsP*) and LIPI-4 (*LM9005581_70009*, *LM9005581_70010*, *LM9005581_70011*, *LM9005581_70012*, *LM9005581_70013* and *LM9005581_70014*); (2) antimicrobial resistance genes (*fosX*/*lmo1702*, *lin*/*lmo0919*, *mprF*/*lmo1695*, *norB*/*lmo2818*, *sul*/*lmo0224*); (3) *inlA* genes carrying internal stop codons.

Comprehensive virulence gene screening using the VFDB showed that all isolates carried the core virulence genes associated with *Listeria* pathogenicity island 1 (LIPI-1), including *prfA*, *plcA*, *hly*, *mpl*, *actA*, and *plcB*. Moreover, isolates belonging to ST619 harbored additional virulence determinants located in LIPI-3 and LIPI-4 (Figure 3), which are often associated with hypervirulent lineages capable of causing central nervous system and maternal–fetal infections. In contrast, all CC9 isolates harbored premature stop codon mutations in the *inlA* gene, indicating comparatively lower virulence potential. Furthermore, the resistance-related genetic profile included the well-characterized core intrinsic resistance gene *fosX*, while *lin*, *sul*, *norB*, and *mprF* were identified as putative resistance-associated genes. All isolates harbored the five aforementioned resistance-related genes (*fosX*, *lin*, *sul*, *norB*, and *mprF*) (Figure 3), which may be associated with corresponding antimicrobial resistance phenotypes.

### 3.3. Yak-Derived L. monocytogenes Isolates Showed Multidrug Resistance and Enhanced Biofilm Formation

**Figure 4:**
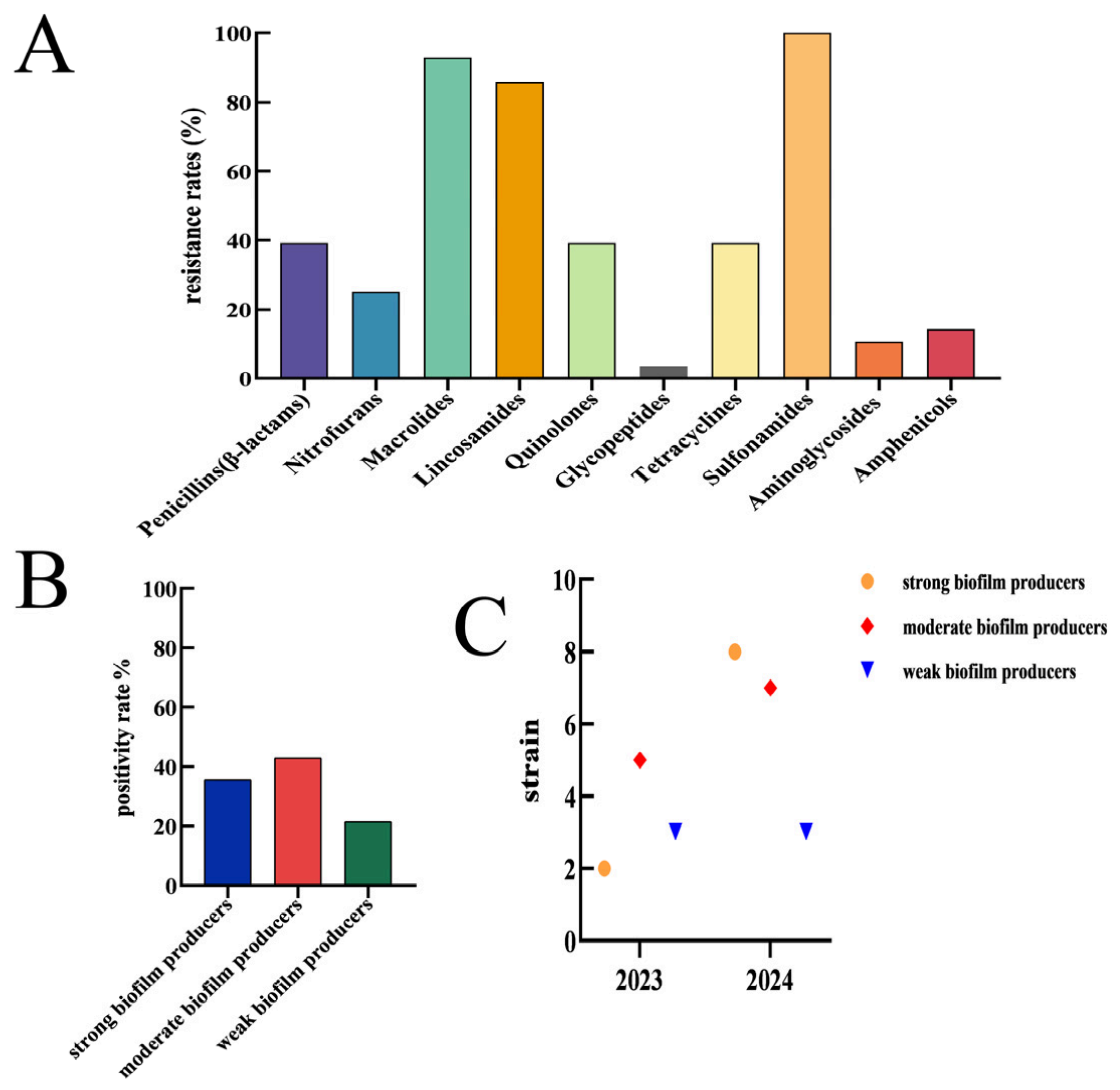
Phenotypic characterization of the isolates. (**A**) Antimicrobial resistance rates of yak meat-derived isolates to clinically common antimicrobial agents. (**B**) Proportions of yak meat-derived isolates with different biofilm-forming capacities. (**C**) Distribution of yak meat-derived isolates by sampling date.

All 28 isolates demonstrated varying degrees of resistance to 10 clinically common antimicrobial agents. High resistance rates were detected for Sulfonamides (Trimethoprim-sulfamethoxazole, 100.00%, 28/28), Macrolides (Erythromycin, 92.86%, 26/28) and Lincosamides (Clindamycin, 85.71% 24/28). Lower resistance was found for Quinolones (Ciprofloxacin, 39.29%, 11/28), Tetracyclines (Tetracycline, 39.29%, 11/28), Penicillins (Ampicillin, 39.29%, 11/28), Nitrofurans (Nitrofurantoin, 25.00%, 7/28), Amphenicols (Chloramphenicol, 14.29%, 4/28), Aminoglycosides (Gentamicin, 10.71%, 3/28), Glycopeptides, (Vancomycin, 3.57%, 1/28) (Figure 4A).

All 28 isolates were capable of forming biofilms, although the degree of biofilm formation varied significantly among STs. Based on OD570 values, 10 isolates (35.71%) as strong biofilm producers, 12 isolates (42.86%) were classified as moderate biofilm producers, and 6 isolates (21.43%) as weak biofilm producers (Figure 4B). Among all tested isolates, ST619 isolates formed relatively high levels of biofilm biomass. This result suggests their potential for persistence in food-processing environments. With respect to the time of isolation, the distribution of biofilm-forming capacity among isolates was generally consistent across different years. However, isolates demonstrating strong biofilm-forming ability were predominantly recovered in 2024, implying a potential trend of enhanced persistence capacity in recent strains (Figure 4C).

### 3.4. Genomic Characteristics of the Representative Isolate from Yak Meat

**Figure 5:**
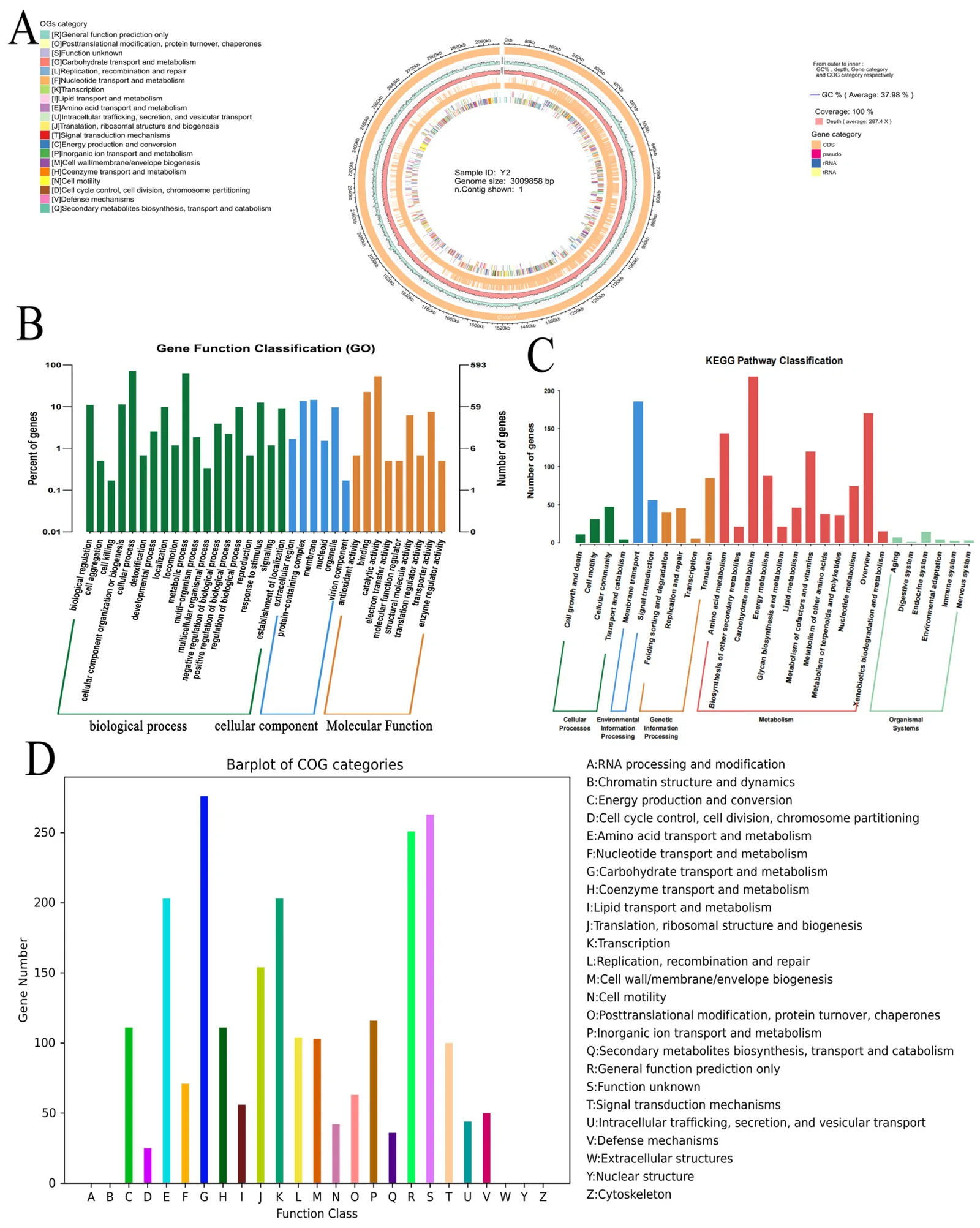
Genome annotation of the representative *L. monocytogenes* Y2. (**A**) Circular genome annotation map of Y2. (**B**) GO functional enrichment analysis. (**C**) KEGG pathway annotation of gene metabolic functions. (**D**) COG functional classification of annotated genes.

The complete genome of representative isolate Y2 (ST619, serotype 1/2b) consisted of a circular chromosome of 3,009,858 bp in length with a GC content of 37.98%, containing 3036 coding sequences (CDSs), 67 tRNAs, and 18 rRNAs (Figure 5A). Genome island prediction identified four genomic islands and two prophage regions. GO enrichment analysis annotated a total of 2058 genes in the representative strain Y2, encompassing the three GO categories: cellular component (CC), biological process (BP), and molecular function (MF). Genes associated with cellular processes, cell membrane components, and catalytic activity were the most abundant (Figure 5B). Functional annotation using the KEGG database showed that most genes were involved in carbohydrate transport and metabolism (9.8%), amino acid transport and metabolism (9.1%), and cell wall biogenesis (8.3%) (Figure 5C). COG analysis yielded consistent results with the aforementioned functional annotations. Carbohydrate transport and metabolism was the most enriched functional category, with the highest number of annotated genes (Figure 5D).

### 3.5. Genetic Divergence Characteristics of Strain Y2 from Relevant Reference Isolates

**Figure 6:**
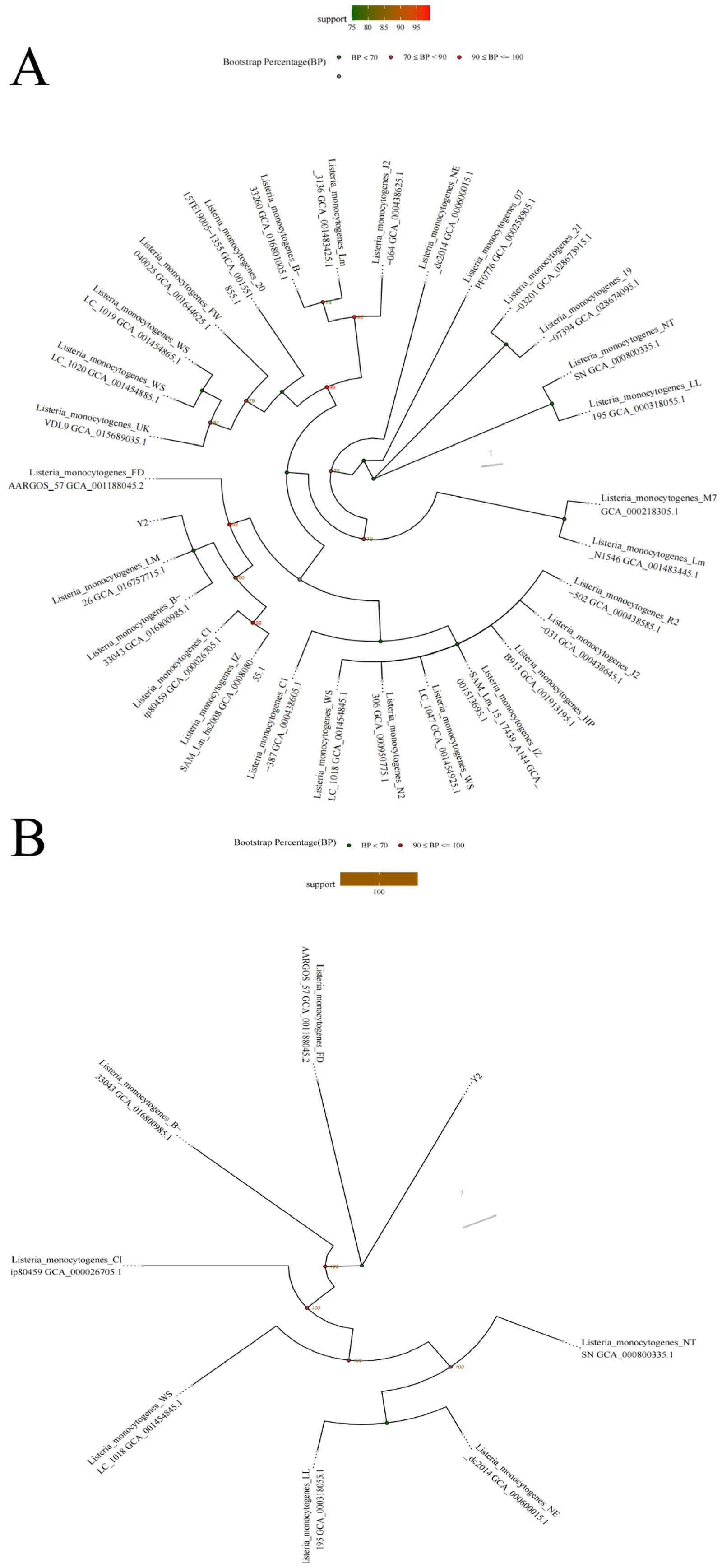
Phylogenetic analysis of the representative *L. monocytogenes* Y2. (**A**) *16S rRNA*-based phylogenetic tree comparing Y2 with clinical isolates. (**B**) Whole-genome SNP analysis of strain Y2 in comparison with clinical isolates.

To explore the evolutionary relationships of the Y2 isolate, 30 *L. monocytogenes* genomes with complete annotations were retrieved from the NCBI database for comparison (Supplementary File S2). 16S rRNA-based phylogenetic analysis indicated that strain Y2 and all reference isolates clustered within a single clade of *Listeria monocytogenes* (Figure 6A). Phylogenetic analysis based on SNP revealed that strain Y2 and reference *L. monocytogenes* isolates represented by GCA_001188045.2, GCA_016009985.1 and others clustered within the same species clade (Figure 6B).

At the strain level, however, substantial genetic divergence exists between Y2 and all reference isolates, with no close phylogenetic clustering detected.

## 4. Discussion

This study is the first comprehensive molecular investigation of *Listeria monocytogenes* in yak meat from Nyingchi, Xizang, China. We found a contamination rate of 13.08%, which is consistent with the levels reported in retail meat across other Chinese provinces (10%~15%) [32]. However, the detection rate in this study is higher than those reported in previous studies conducted in Norway (Europe) and Ethiopia (Africa) [8,33]. These differences may stem from variations in slaughtering hygiene and meat-handling practices. The predominant serotypes (1/2a, 1/2b, and 1/2c) align with major clinical and food-associated strains found globally. Notably, while ST8 and ST9 are common persistent clones in food environments, the identification of ST619 is concerning. Unlike the more common ST9, ST619 has been increasingly linked to listeriosis, suggesting that yak meat may serve as a potential transmission vehicle for *L. monocytogenes* strains with clinical relevance [34,35].

Phylogenetic analysis (MLST and cgMLST) revealed that isolates from different batches clustered into the same clades (ST8 and ST619). This indicates that specific *L. monocytogenes* clones can persist in the yak meat production chain for extended periods. Similar long-term persistence of ST8 has been documented in salmon and poultry processing plants in Europe [36]. The recovery of identical ST strains from different retail stalls suggests the existence of a potential common contamination source. Sites such as floor drains and processing equipment provide stable habitats. *L. monocytogenes* can survive routine sanitation, which may lead to sustained cross-contamination of yak meat.

All isolates carried core virulence genes (LIPI-1), but the presence of LIPI-3 and LIPI-4 in ST619 strains is a major public health concern [37,38,39]. LIPI-3, which encodes listeriolysin S (LLS), is typically found in lineage I strains and enhances survival in the host gut [40,41]. LIPI-4 is a known neuro- and placental-tropic cluster [42]. The coexistence of these islands makes ST619 more hazardous than common food isolates like ST9. The ST619 isolates exhibited the highest average biofilm formation capacity, indicating their strong long-term survival capability in food-processing environments. Compared with strains of other sequence types (STs), the biofilm-forming trait of ST619 accounts for its environmental persistence and repeated contamination detected in this sampling, and highlights the need for targeted cleaning and disinfection strategies in meat processing facilities in Tibetan areas.

Complete genome analysis of the ST619 isolate (Y2) revealed various prophages and genomic islands related to environmental adaptation. The enrichment of genes for carbohydrate metabolism and cell wall integrity likely supports *L. monocytogenes* growth under the cold, nutrient-limited conditions of high-altitude storage. The identification of multiple genomic islands and prophages implies frequent horizontal gene transfer among these strains. Importantly, the ST619 sequence type has been reported in isolates from multiple regions worldwide, including China and European countries. This indicates that ST619 is not a geographically restricted clone, but a widely distributed sequence type with potential public health relevance. The ST619 isolates in this study carry multiple virulence-associated genes at the genomic level. With the expanding cold-chain distribution of yak meat to other regions, attention should be paid to the potential foodborne transmission risk of this pathogen to wider populations. Against the backdrop of the ongoing expansion of cross-regional cold-chain distribution, the findings of this study provide fundamental baseline evidence for advancing food safety across the full yak meat supply chain. Specifically, they offer theoretical support for optimizing key links including slaughter hygiene, raw meat handling, retail environmental hygiene, cold-chain management, and cross-contamination control, with the aim of mitigating *Listeria monocytogenes* contamination risks.

This study was limited by its sampling scope, as isolates were collected from the specific region and timeframe. Future research should include a wider geographic area and longitudinal sampling to better understand *L. monocytogenes* dynamics. Additionally, transcriptomic studies are needed to clarify how these strains adapt to the unique high-altitude, low-oxygen environment of Xizang, and phenotypic validation should be conducted to confirm the virulence and environmental tolerance traits predicted by genomic analysis. Nevertheless, our findings provide a critical baseline for microbiological surveillance in yak meat and underscore the need for improved hygiene standards from slaughter to retail.

## 5. Conclusions

This study characterizes the genomic profiles of *Listeria monocytogenes* isolated from retail yak meat in Nyingchi, Xizang, China. The dominant ST619 harbors intact LIPI-1, LIPI-3 and LIPI-4 pathogenicity islands with strong biofilm-forming capacity, traits that may enable its persistence in processing environments and subsequent cross-contamination. Concurrent detection of ST619 and antimicrobial resistance loci implies potential animal–human exposure concerns, highlighting the necessity of standardized hygiene management across the entire yak meat supply chain. Sampling of the current study focused only on retail yak meat within a single region, with no matched isolates collected from abattoirs or surrounding environments. Virulence potential was deduced from genomic signatures, which requires further phenotypic assays for confirmation. Even so, these data deliver fundamental molecular evidence for plateau-wide *L. monocytogenes* surveillance. Future monitoring should expand sampling coverage and adopt high-resolution genomic typing to trace high-risk clones and optimize regional strategies against foodborne listeriosis.

## Figures and Tables

**Table 1:** List of antibiotic discs used in this study.

Antimicrobial Class	Antimicrobial Agent	Zone Diameter Breakpoints (mm)			Guideline Used	Potency
		Resistant	Intermediary	Sensitive		
Penicillins(β-lactams)	Ampicillin	<16	-	≥16	EUCAST v. 16.0	2 µg
Nitrofurans	Nitrofurantoin	≤14	15–16	≥17	M100 (*Staphylococcus* spp.)	300 µg
Macrolides	Erythromycin	<25	-	≥25	EUCAST v. 16.0	15 µg
Lincosamides	Clindamycin	≤14	15–20	≥21	M100 (*Staphylococcus* spp.)	2 µg
Quinolones	Ciprofloxacin	≤15	16–20	≥21	M100 (*Staphylococcus* spp.)	5 µg
Glycopeptides	Vancomycin	≤14	15–16	≥17	M100 (*Staphylococcus* spp.)	30 µg
Tetracyclines	Tetracycline	≤14	15–18	≥19	M100 (*Staphylococcus* spp.)	30 µg
Sulfonamides	Trimethoprim-sulfamethoxazole	<29	-	≥29	EUCAST v. 16.0	25 µg
Aminoglycosides	Gentamicin	≤12	13–14	≥15	M100 (*Staphylococcus* spp.)	10 µg
Amphenicols	Chloramphenicol	≤12	13–17	≥18	M100 (*Staphylococcus* spp.)	30 µg

## Data Availability

Genome sequences information is available in https://www.ncbi.nlm.nih.gov/nuccore/ (accessed on 7 January 2026), under the GenBank ID CP184647.1.

## Supplementary Materials

The following supporting information can be downloaded at: https://www.mdpi.com/article/10.3390/ani16172729/s1. File S1: 28 Samples of Yak meats with Related Information on Their Origin. File S2: Thirty Listeria monocytogenes genomes with complete annotation information. File S3: Y2-Genomic Island. File S4: Y2-prophage.
